# AI-enabled resilience modeling for brain health

**DOI:** 10.3389/fmmed.2025.1671337

**Published:** 2025-10-01

**Authors:** Andrew Wister, Elizaveta Pinigina, Jie Liang, Igor Linkov

**Affiliations:** ^1^ Gerontology Research Centre & Department, Simon Fraser University, Vancouver, BC, Canada; ^2^ Tor Intelligence, Boston, MA, United States; ^3^ AltumView Systems Inc., Simon Fraser University, Vancouver, BC, Canada; ^4^ Agricultural and Biological Engineering, University of Florida, Gainesville, FL, United States; ^5^ Engineering and Public Policy at Carnegie Mellon University, Pittsburgh, PA, United States

**Keywords:** brain health, AI-models, resilience, research agenda, challenges

## Abstract

One development in the growing field of Alzheimer’s Disease and related neurological disorders (ADRD) is the consideration of brain resilience, the ability to respond to and recover from adversity, which builds on a growing literature on the role of lifestyle behaviours in ADRD prevention and response. This paper reviews definitions of ‘brain health’ and integrates these with innovations in resilience system models applied to ADRD. Based on a socio-ecological framework that links physiological, behavioral, economic, and social determinants of mental health, we propose a unified model of resilience and aging in this field. We contend that applications of a resilience analytical approach to brain health require innovation in Artificial Intelligence (AI) to harness the full potential of immense interdisciplinary data mining opportunities. These include: development of digital twins, precision health analytics, AI sensors, and Multimodal Large Language Models (MLLM), knowledge graph technologies, and cognitive/decision science modeling. We apply this model to research and clinical examples to elucidate its potential value, requirements, risks, and challenges in developing new research agendas.

## Introduction

The field of brain health and aging has grown exponentially in parallel with the rise in Alzheimer’s Disease and related neurological disorders (ADRD), coupled with interest in understanding trajectories of cognitive decline (Kleiman et al., 2022). This has led to a focus on prevention and treatment, which as a prerequisite requires a deeper understanding of all forms of cognitive impairment from an interdisciplinary perspective that transcends genetic biomarkers and clinical tests (Hachinski and Avan, 2022). The concept of resilience is a recent addition to aging and brain health initiatives, which offers an interdisciplinary conceptual framework to understand how and why some individuals prevent, adapt to, and/or recover from ADRD better than others (e.g., Cassidy and Cassidy, 2019; Dutcher, 2023; Hachinski and Avan, 2022; Kalisch et al., 2015; Kleiman et al., 2022; Rothman and Mattson, 2013; Wister et al., 2022). This approach is embedded in a socio-ecological framework, recognizing individuals as nested within interconnected ecosystems of physiological, behavioral, economic, and social determinants of brain health (Klasa et al., 2021; Wister et al., 2022).

This complex systems perspective examines interdependent biological and physiological aging mechanisms alongside broader social determinants data, and as a result, requires a pathway for analyzing the complex interplay of aging processes on a mass scale. We argue that a resilience strategy needs to blend the best practices in dementia data handling utilizing current development in AI, such as AI sensors and Multimodal Large Language Models (MLLM), knowledge graph technologies, and cognitive/decision science modeling, enabling groundbreaking analytical solutions for brain health to harness the immense AI and data mining capabilities necessary for such a complex cross-disciplinary analysis (Busch et a., 2025; Meng et al., 2023).

## Current state of brain health science and practice

The current scientific discourse surrounding brain health predominantly revolves around understanding the mechanisms of cognitive performance and its decline with aging. Central to this discourse are concepts such as cognitive reserve, brain resilience, and compensatory mechanisms, which are posited to influence the trajectory of cognitive aging and vulnerability to neurodegenerative diseases, such as Alzheimer’s Disease and related dementias (ADRD) (Joshi and Galvin, 2022; Stern, 2012). Significant theoretical developments in the field of neurology and cognitive sciences have introduced a variety of additional terms that are crucial to understanding brain health that integrates physiological, psychological, and social components (Kalisch et al., 2015; Wister et al., 2016). Among these, the concept of *brain resilience* is frequently discussed alongside cognitive reserve. This term is typically defined as the brain’s inherent capacity to resist damage due to aging or pathology through the biological robustness of neural pathways that maintain cognitive functions despite various cognitive adversities.

Hachinski and Avan (2022) offer a comprehensive definition that positions brain resilience as “the lifelong, dynamic, interactive, accumulating capacity to resist physical, emotional, and/or social harms.” This definition not only highlights the dynamic and cumulative nature of cognitive resilience but also emphasizes the necessity of addressing the broad spectrum of influence—from molecular to societal—that affect cognitive health over an individual’s lifespan. Further, optimal brain health represents a holistic term integrating aspects of resilience and reserve, proposing a state in which the brain operates at its best capacity, maintaining high function and delaying degenerative processes as long as possible.

These conceptualizations are instrumental in framing research agendas; however, they often remain confined within specific disciplinary boundaries, focusing heavily on physiological and pathological outcomes (Kalisch et al., 2015). This lens tends to overlook the broader spectrum of aging processes that impact cognitive health, thus necessitating a more integrated approach that considers the complex interplay of biological, psychological, and social factors (Wister et al., 2022). Additionally, while the existing models of brain health are valuable for outlining the basic frameworks of cognitive functionality and resilience, they exhibit several critical gaps. Empirical robustness is notably lacking, as many of these models are predominantly theoretical and do not have comprehensive empirical support for measuring brain health resilience through aging or interventions, especially in longitudinal studies that can genuinely capture their dynamic nature. Furthermore, interdisciplinary integration is often lacking. Theoretical models of brain health rarely cross disciplinary boundaries, which limits their applicability in real-world settings where biological, psychological, and social factors intersect. There is also an absence of a life course perspective that accounts for the complex effects of these components over long-term life trajectories, such as the cumulative effects of poverty, or the timing of stressful events such as the COVID-19 pandemic.

To bridge these gaps, there is an emerging consensus on the need to empirically evaluate “resilience” as it pertains to brain health (Cassidy and Cassidy, 2019; Hachinski and Avan, 2022; Joshi and Galvin, 2022: Kalisch et al., 2015; Kleiman et al., 2022; Rothman and Mattson, 2013). In this context, resilience does not merely refer to the capacity to recover from adversity but also includes the ability to maintain stable, high levels of cognitive and neurological functioning amid the complex interplay of aging processes. Technological and analytical advances, such as machine learning (ML), AI (especially powerful AI sensors and MLLMs), and virtual reality, can play pivotal roles in collecting multimodal data and deciphering the complex patterns and interactions among the myriad of factors affecting brain health. These tools can help identify potential interventions tailored to individual profiles derived from broad-based and inclusive big data sources that evaluate brain health resilience from acute or chronic stressors arising from various origins (e.g., social, health, and others), thus enhancing the precision and effectiveness of preventative and therapeutic strategies.

Indeed, these innovations are already employed to assist in ADRD diagnostics, research, and patient care in practice (Yang and Mohhamed, 2020). For example, in past research, AI technologies have been used to assist in the analysis of neuroimagery as well as novel biomarkers in blood linked with AD; and virtual reality models have been incorporated into facilitating singing exercises within Alzheimer impacted patient groups (Kiss et al., 2023). Privacy-preserving AI sensors have been used to monitor the physical activities of older adults and patients, to collect activity statistics, and to notify caregivers in case of emergencies such as falls. This ensures the safety of older adults living alone, especially those with ADRD, and provides a powerful tool for the analyses of the activity data collected, evaluate the conditions of the older people and patients, and design interventions and treatment plans accordingly (Agarwal et al., 2022; Liang et al., 2024). AI-powered robotics have also been used as companions to older adults with ADRD to alleviate their loneliness (Broadbent et al., 2024).

Despite the effective use of these innovations, there is currently a limited application of these technologies to interventions specifically geared at resilience building, such as in the case of the AAL RESILIEN-T project that employed ML and AI algorithms to optimize data extraction and sensor calibration aimed at building patient resilience (Cassacia et al., 2019). The limited integration of AI and ML technologies with resilience-specific approaches geared at AD creates a space for the propagation of the framework and applications such as the one discussed in this paper. With the increased focus on brain resilience in scientific discourse, there is a need for practice to reflect the theoretical backbone within the AD research field. The rapid development of AI, especially MLLM and AI sensors, allow them to be used to develop the resilience models, collect more data, and improve the performance of the resilience building systems. However, the design of these systems should also consider the various computational requirements, challenges, and potential risks of utilizing AI and MLLM, to ensure reliable, safe, and ethical AI-assisted solutions (Afkanpour et al., 2024; Atf et al., 2025; Busch, 2025; Stempfle et al., 2025).

## Conceptualizing resilience in the context of brain health

Many definitions of resilience exist across a variety of disciplines (e.g., psychology, sociology, gerontology, biology, physiology, environment, etc.) over several decades with foci on different adversities (e.g., mental health, life crises, multimorbidity, natural disasters, etc.); yet they share the idea that [it] is a strength-based framework that explicates the ability of an individual, community, or system to adapt, recover and/or grow from a type of adversity (Fry and Keyes, 2010; Masten, 2007; Resnick et al., 2019; Wister et al., 2016; Wister and Cosco, 2020). A family of resilience theoretical models has appeared in the literature that either applies to a particular adversity or is generic (Luthar and Brown, 2007; Masten, 2015; Resnick et al., 2019; Windle, 2012; Wister et al., 2022). One model, the Unified Model of Resilience and Aging (UMRA) begins with the disruptive event (a pandemic, illness, personal loss) and its impact on the individual followed by possible activation of internal resources and external resources (e.g., optimism, support from family, friends or organization, policy environment), and the potential for reintegration and even growth resulting from the resilience process.

Underlying the UMRA and other models is the life-course dimension (time, place and epigenetics) situating experience. The UMRA also includes four system-level (organizational) functions used by the National Academy of Sciences Resilience Model: 1) Planning for adverse events requires reductions in risk in response to an identified threat. 2) Absorption of stressors and outcomes associated with adversity is necessary to initiate resilience through recovery and adaptation. 3) Recovery occurs through various forms of short and long-term strength-based resilience. 4) Adaptation relates to changes in the system to promote future resilience.

Several core elements of resilience theory can be applied to a brain health research agenda. First, the type, intensity, and context of the adverse condition or event is a central component of a resilience framework. It is well-known that Alzheimer’s and related dementia follow a pathogenetic trajectory that is slow in progression, sometimes decades in length. If prevention is to be successful, upstream and life-course situated health promotion, prevention, and public health strategies will be most effective but also require habitual repetition, such as routinized healthy lifestyle behaviours. The question is: how do we identify modifiable and non-modifiable risk factors and levels at an early enough stage to target and tailor resilience-enhancing strategies?

Thus far, genetic markers can identify one level of risk but are only one component of what has become understood as a highly complex causal structure. Resilience models are well-suited to incorporate the interdisciplinary multifactorial pathways of ADRD (i.e., genetic, behavioural, and environmental levels). This requires bringing together researchers from different disciplines and applied/basic research environments for innovation and intervention/treatment breakthroughs. Resilience models emphasize a strength-based approach to prevention that can build brain health throughout life, rather than attempt to treat the condition once it has progressed to a more challenging treatment stage. However, identifying the most treatable moments in the multifaceted life-courses of individuals, to date, remains a conundrum. Further, many modifiable preventive behaviors, such as healthy levels of physical activity; obesity, diet and nutrition; reduced alcohol consumption; good sleep patterns; decreased head injuries; low stress; lower exposure to pollution; improved mental activity; and other healthy lifestyles are typically examined independently, although research is demonstrating that it is their combined and cumulative effects that are most influential for prevention and treatment of ADRD (Livingston et al., 2020; Reuben et al., 2024; Son et al., 2025). For instance, in an examination of clustering of predictors of 3-year cognitive decline using data from the Canadian Longitudinal Study of Aging revealed that dyad combinations of hearing loss and physical inactivity were linked to declines in cognition; hearing loss, physical inactivity, and hypertension for the triad; and hearing loss, physical inactivity, hypertension, and sleep disturbance for the tetrad (Son et al., 2025).

Applying resilience models within brain health research with an added layer of AI algorithms can potentially advance our understanding of ADRD by identifying modifiable risk factors and critical periods for intervention, and thus, delay or prevent the onset of clinical symptoms. For instance, are there particular times in a person’s life in which physical activity, sleep, mental exercises, or nutritional interventions result in maximum effects? Moreover, understanding the interactions between genetic predispositions and lifestyle factors can guide personalized medicine approaches that optimize brain health across the lifespan. One of the primary hurdles is the complexity of causal structures involved in cognitive aging. The multifactorial pathways that influence cognitive resilience and decline encompass a broad array of biological, psychological, and social variables, each interacting in non-linear ways that are difficult to predict and model (Reuben et al., 2024). The sophisticated models required for this analysis must be capable of handling these complex interactions to provide insightful and actionable findings. These models are essential not only for understanding the interdependencies of these variables but also for developing interventions that can robustly support cognitive health across the lifespan. Moreover, cost and feasibility limitations in clinical intervention design that combine multiple factors into different treatment groups (e.g., genetic profile, pharmacology, physical activity, nutrition, smoking, alcohol, stress, etc.) hamper treatment development. Designing research that can effectively isolate and evaluate the effects of intertwined factors requires substantial financial resources, advanced technological tools, and a multidisciplinary approach that often goes beyond the conventional boundaries of neuroscientific research. The logistical and financial implications of such comprehensive studies make it difficult to regularly implement them at a scale that would provide statistically meaningful results. This challenge is compounded by the need for longitudinal data, which are crucial for assessing the progression of cognitive resilience and decline over time but are expensive and complex to collect and analyze. This creates an opportunity for the utilization of ML and AI algorithms and AI products for comorbidity identification and integration of socioeconomic factors due to these technologies’ capacity for large data set analysis and extensive processing power (Alsaleh et al., 2023). For example, using low-cost yet powerful embedded AI chips and the latest computer vision algorithms, it is possible for an AI sensor to detect people in a room in a contactless way, and extract the skeleton of the human bodies represented by some key joint points in real time (Agarwal et al., 2022; Liang et al., 2024). The skeleton data provide valuable information about the person’s activity and health condition, and the amount of skeleton data is much less compared to the raw video data. Skeleton data can be saved on the server for longitudinal activity analysis, using more sophisticated AI algorithms, such as MLLM, which can be useful for various medical tasks, including the study of resilience associated with brain health. However, the utilization of AI approaches to investigate brain health causes and treatments must address these same challenges of disentangling highly complex processes (Son et al., 2025).

There are also critical ethical and social considerations that must be navigated carefully. Research and interventions aimed at enhancing resilience must be designed with a keen awareness of ethical standards and socio-economic equity to ensure that their benefits are accessible across different segments of society, including the most vulnerable populations. It is imperative that these initiatives not only target high-risk groups but also accommodate diverse community needs in a manner that promotes fairness and inclusivity. These ethical concerns center on patient privacy and informed consent protocols. AI relies on vast amounts of information to function, necessitating access to patients’ personal medical data (Horn, 2001). As a result, hospitals and research institutions must make significant efforts to uphold patient privacy and implement robust cybersecurity measures. Patient consent is also a critical tenet in the implementation of AI technologies for medical research. However, current informed consent protocols may not fully account for the application of data mining in AI models (Kotsenas et al., 2021). The integration of AI-specific disclosures and the strengthening of patient information repositories will be essential for the ethical implementation of these technologies. Each of these challenges demands careful consideration from healthcare providers and collaborative efforts across various fields of research and practice to integrate updated ethical considerations for AI innovations. Addressing these ethical considerations involves complex consent processes, privacy issues related to data handling, and the implementation of interventions that are sensitive to the cultural and social dynamics of target communities.

Each of these challenges demands innovative technical solutions and collaborative efforts across various fields of research and practice. By addressing these hurdles head-on, the scientific community can advance our understanding of resilience as it pertains to brain health and move closer to developing effective strategies that mitigate cognitive decline and enhance the quality of life for aging populations worldwide. These efforts are crucial in paving the way for preventive health measures and therapeutic interventions that are both scientifically sound and socially responsible.

Significant progress has been made in considering patient privacy and designing informed consent protocols when deploying AI products. For example, when computer vision technology is used for activity analysis, the skeleton representation of human activity not only reduces the amount of data transmitted, but also protects the privacy of the person being monitored, allowing it to be used in sensitive areas such as bedrooms and bathrooms (Agarwal et al., 2022; Liang et al., 2024). These technologies have been adopted by individual Amazon customers in United States and Canada, as well as senior care facilities in various countries, including United States, Canada, Australia, Japan, and some European countries, where consent protocols are developed to explain how the system works, what kind of data are collected and transmitted to the server, and who has access to these data.

Privacy concerns, however, remain a major impediment for AI applications, compounded by critical issues regarding model generalizability and accuracy degradation when algorithms are deployed in novel contexts. Recent healthcare data breaches have affected over 176 million patients in the United States, with most incidents resulting in employee negligence rather than external threats, underscoring the vulnerability of patient information in digital health systems (Theodos and Sitting, 2020). Maintaining robust data privacy during AI application deployment necessitates the integration of advanced blockchain technologies and secure multi-party computation protocols to preserve the integrity and confidentiality of large datasets containing sensitive patient information (Williamson and Prybutok, 2024).

The degradation of analytical quality when AI models are applied across diverse healthcare settings also presents a particularly pressing concern, especially considering deployment between different regions or countries where clinical procedures, medical equipment, and fundamental healthcare philosophies may differ substantially (Yang et al., 2024). Empirical evidence demonstrates that different healthcare settings vary significantly in terms of unobserved confounders, protocols, deployment environments, and temporal drift, with models developed in high-income countries often experiencing substantial performance degradation when applied to low-middle income country contexts (Yang et al., 2024). Researchers and implementers of AI technologies must therefore adopt deliberate governance approaches to account for contextual and systemic variations when implementing AI models for Alzheimer’s patients across different geographic regions and clinical environments, potentially benefiting from the development of multiple localized AI models rather than relying on a singular, universal algorithmic solution.

One way to address this challenge is to fine-tune or adapt the general-purpose AI or MLLM models using the training data obtained from the current application setting (Cheng et al., 2025; Du et al., 2024; Treder et al., 2024). This approach requires much less computational resource than re-training a general-purpose model.

Another risk or challenge of using MLLM is hallucination, which is a limitation of MLLM that can undermine trust, spread misinformation, and pose risks in critical applications. One way to reduce hallucinations in MLLM for ADRD treatment is to employ Retrieval-Augmented Generation (RAG) to ground responses in specific, verified ADRD knowledge bases, and adjust model generation parameters like temperature to favor factual accuracy over creativity. Additionally, implement post-processing steps such as rule-based filtering and human review to catch and correct any remaining incorrect outputs, while prompt engineering techniques like few-shot prompting can also guide the model toward accurate, domain-specific responses (Treder et al., 2024).

Environmental considerations present an additional dimension of concern, as AI systems contribute to environmental degradation through substantial resource consumption. Digital health technologies, including AI applications, require significant quantities of raw materials for device production, while data storage demands vast amounts of electricity for server operation and cooling systems, which causes the environmental footprint to go beyond hardware production to encompass energy-intensive computation processes. (Thompson, 2021; George et al., 2023). Projections suggest that AI could account for 1% of global electricity consumption if current utilization trends continue at their present trajectory (Gaetani et al., 2024). Proposed mitigation approaches include purchasing carbon offsets to compensate for the negative environmental impacts of AI server infrastructure, alongside encouraging sustainable AI development through strategic triage, prioritizing deployment for novel symptom analysis, complex diagnostic scenarios, and preventative health interventions over applications in elective treatment contexts (Richie, 2022). These considerations emphasize the necessity for comprehensive environmental impact assessments in healthcare AI implementation strategies. Although the training and inference of many existing AI and MLLM models require expensive GPU resources, the research and industry communities are developing many new technologies, leading towards lower-cost and high-performance AI and MLLM. This involves novel designs in chiplet architectures, more efficient AI models, and hardware-software co-design, therefore fostering performance gains beyond traditional Moore’s Law’s transistor-doubling pace (Yazdanbakhsh, 2025). For example, the latest MiniCPM-V model can achieve GPT-4V level MLLM performance from OpenAI on mobile phones (Yao, 2024).

## AI and advanced data analytics techniques to operationalize resilience

The endeavor to operationalize resilience in the context of brain health critically depends on the integration of advanced technologies such as AI and comprehensive data analytics. These technologies offer the transformative potential to overcome traditional barriers in dementia and Alzheimer’s research, characterized by reliance on either purely quantitative or qualitative data frameworks that seldom intersect seamlessly. Previous methodologies display considerable limitations in flexibility, adaptability, and precision, particularly when navigating complex, multifaceted datasets commonly missing substantial data portions essential for comprehensive analysis (Son et al., 2025; Windle et al., 2011). However, recently the rapid development in the field of MLLM has opened up many exciting opportunities (Cheng et al., 2025; Treder et al., 2024; Du et al., 2024).

AI and machine learning algorithms present unprecedented advantages in their ability to process and analyze large datasets that include a variety of biomarkers associated with aging and dementia risk. Conventional research tools often fail to account for the temporal dynamics of these biomarkers or effectively combine quantitative with qualitative data.

The latest AI methodologies represented by large language models (LLM) excel in this realm. All existing LLM technologies such as ChatGPT from OpenAI, Gemini from Google, Llama from Meta, and Claude from Anthropic, are based on the transformer technology (Treder et al., 2024; Vaswani et al., 2017). Transformer excels at analyzing longitudinal data for a few reasons: 1) LLMs are able to uncover trends and patterns across long periods; 2) LLMs are adept at understanding contextual information; 3) LLMs can help fill in gaps in longitudinal data, either by predicting missing values, or forecasting based on historical trends; 4) LLMs can offer multidisciplinary insights, because LLMs have been applied to diverse data sources, and they can incorporate perspectives from different fields; 5) LLMs can easily integrate multimodel data and becomes a power multimodel (MLLM), which is crucial for the brain health studies of ADRD.

Therefore LLMs can drive the capacity for more nuanced analyses that can integrate diverse data types, including structured, unstructured, and semi-structured data. This integration is crucial for developing more accurate and dynamically adaptable models of brain health that can forecast and respond to the progression of cognitive decline with greater precision.

**FIGURE 1:**
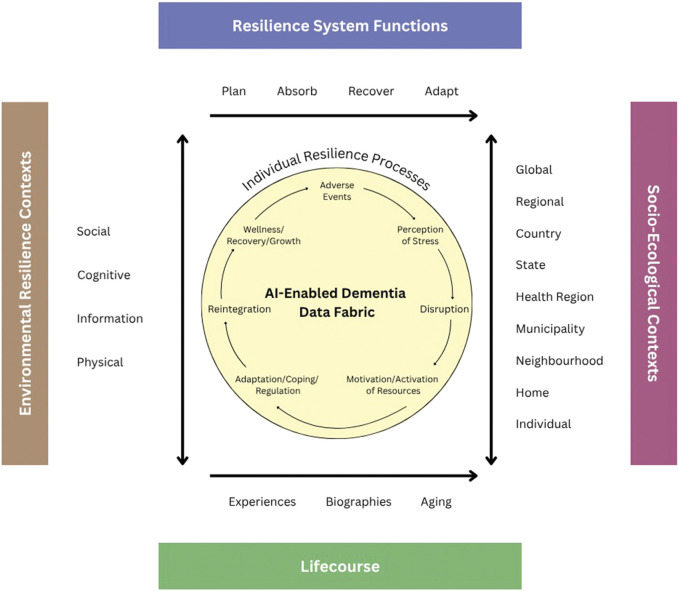
A unified Model of Resilience and Aging.

A significant advancement offered by AI is its capability to synthesize information across various data streams to provide a holistic view of an individual’s cognitive health. This process involves the use of sophisticated decision analysis and cognitive modeling techniques that capture and reflect user needs and values, thereby personalizing precision treatment plans. Furthermore, AI-driven analytics facilitate the development of self-driven hypothesis testing and expansive data exploration practices that were previously unattainable with traditional analytical tools. Through these methods, AI helps elucidate the intricate interplay between brain function and various resilience factors within the socio-economic and environmental context—commonly referred to as brain capital. One way to frame this analytic approach is the Unified Model of Resilience and Aging (UMRA, Figure 1) (see Wister et al., 2022 for full discussion). This model serves as a pivotal framework in the application of advanced analytical capabilities, particularly integrating AI to enhance the empirical evaluation of resilience within brain health research. It adopts a life-course perspective that meticulously incorporates resilience functions such as planning, absorption, recovery, and adaptation—these are well-endorsed by the National Academy of Sciences and pivotal in addressing the multi-layered nature of aging and cognitive decline. UMRA contextualizes the resilience processes of individuals within an extensive socio-ecological framework, taking into account the myriad of interactions that influence resilience over an individual’s lifespan (Infurna, 2021). This comprehensive approach not only elevates the relevance of interventions tailored for aging populations but also enhances their applicability by addressing specific socio-ecological dynamics and resilience needs at various life stages.

In operationalizing UMRA, the concept of ‘AI-enabled’ data fabric’ plays a crucial role. It solves two issues of crucial importance for ADRD research: (i) the ability to store large volumes of heterogeneous information, and (ii) quick fusion of information based on different weighting schemes. This integrative tool encompasses a complex network of data entities and relationships that span across diverse data modalities, including medical records, survey and clinical studies, scientific literature, and demographic insights. The utility of AI-enabled data fabric extends beyond mere data storage and standardization; it actively employs advanced ontologies and taxonomies to organize data, while utilizing flexible scripting and natural language processing technologies to transform and harmonize disparate data types into analytically robust formats. Formal data fusion algorithms can utilize tools of Multi Criteria Decision Analysis (MCDA), (Linkov et al., 2021), that allows integration of measurable data points with value systems that clinicians can assign based on the quality and relevance of measures reflective of patients’ value systems. In our previous research, we argue that the use of MCDA in the context of AI can enable interpretation of AI models and provide confidence to both patients and physicians associated with considered treatment alternatives (Linkov and Trump, 2019: Linkov et al., 2020).

The implementation of a robust data integration system via MCDA-enabled data fabric offers substantial benefits. It ensures a seamless and transparent flow of information and supports the integration of multimodal data sources, including medical, genetic, socioeconomic, behavioural and clinical data. This integration is critical for performing harmonized analyses across traditionally isolated datasets, thus allowing for comprehensive assessments that can identify nuanced relationships and patterns significant to brain health. By effectively reconciling terminological and representational discrepancies, AI-enabled data fabric significantly simplifies the navigation through interconnected data entities related to a patient’s historical health records, exposure levels, and potential risk factors, thereby enhancing the precision of hypothesis formulation and evidentiary assessments. This capability is crucial for maintaining the integrity and lineage of data, which is essential for accurately tracing interventions, outcomes, and hypothesis testing across the convoluted landscape of brain health research.

Furthermore, the predictive capabilities of this integrated system are instrumental in anticipating the impacts of potential interventions or environmental changes on patient outcomes. Through the creation of simulated environments and scenarios, researchers and clinicians can proactively test and refine intervention strategies, which prepares the healthcare system to respond efficiently and effectively to emerging challenges. This proactive engagement not only mitigates potential risks but also enhances the adaptability of health interventions, ensuring that they remain effective in the face of new discoveries and evolving patient needs. This dynamic approach to health management underscores the importance of continuous innovation and adaptability in the field of brain health, particularly as it relates to fostering resilience among aging populations.

## Benefits of AI and advanced data analytics to enhance brain health

The current discourse on brain health resilience emphasizes the integration of various conceptual models from neurology, psychology, and socio-ecological disciplines, revealing the complex interplay between genetic, behavioral, and environmental factors. This complexity necessitates a multidisciplinary approach to understand and enhance cognitive resilience effectively. Models such as UMRA facilitate this integration by framing resilience through a life-course perspective that incorporates planning, absorption, recovery, and adaptation phases endorsed by the National Academy of Sciences. Such models are not only pivotal for theoretical advancements but also enhance practical applications in clinical settings, where personalized interventions can be tailored based on comprehensive, data-driven insights.

The application of AI in operationalizing resilience within brain health research represents a paradigm shift towards more predictive, personalized, and preventative healthcare. AI algorithms and machine learning models afford the capability to process and analyze large-scale, heterogeneous data sets, thereby identifying patterns and interactions that are not readily apparent through conventional statistical methods. This capability is crucial for developing dynamic models of brain health that adapt to new data inputs and evolve in response to emerging research findings and clinical practices.

Despite these advancements, significant challenges remain in the realm of data integration. The AI-enabled data fabric approach, while robust in theory, requires meticulous implementation to ensure the integrity, accessibility, and compatibility of data across various sources and formats. The standardization of data using ontologies and taxonomies, coupled with the flexible processing capabilities of natural language processing and AI scripts, is essential for the effective operationalization of resilience models. Moreover, data fusion methodologies in which subjective judgement and values need to be integrated are not developed, but integration of MCDA may help in both transparent fusion of heterogeneous information and visualizing confidence associated with AI predictions. These processes are resource-intensive and require ongoing adjustments to align with new scientific evidence and technological developments. Further, the deployment of AI and data analytics in brain health necessitates careful consideration of ethical, legal, and social implications. Issues such as data privacy, consent for data use, and the potential for algorithmic bias must be addressed to ensure that these technological solutions do not inadvertently perpetuate disparities in healthcare access or outcomes.

## Challenges of handling incomplete, irregular or missing data

Incomplete, irregular, or even missing data are quite common in many healthcare settings. If not handled properly, they can cause the MLLM to generate unsafe or misleading recommendations. Some approaches can be employed to mitigate the incomplete or missing data, such as imputation, missing-aware training, RAG, and human-in-the-loop validation. A careful combination of these approaches is usually required, based on a deep comprehension of the data being analyzed (Afkanpour et al., 2024; Atf et al., 2025; Busch, 2025; Stempfle et al., 2025).

## Data case example: the Canadian Longitudinal Study on Aging

One case example is the use of the Canadian Longitudinal Study on Aging (CLSA). The CLSA is a national study that started in 2010 with data collected every 3 years, starting with Baseline data collected among over 51,338 Canadians aged between 45 and 85 when recruited. The CLSA data contain a wide range of information related to demographic background, physical and mental health, family and social life, employment and retirement situation, etc. of participants (for detailed information, see Raina et al., 2019). The CLSA participants are comprised of two cohorts, the Comprehensive cohort and the Tracking cohort. Participants from the Comprehensive cohort were randomly selected from the population (within age/sex strata) within 25 km (50 km for lower population density areas) of the established 11 CLSA data collection sites, and data were collected on-site and via a home interview and includes physiological data (blood for biomarkers, urine, bone density, hearing, sight, etc.), cognitive status, health profile, and a face-to-face interview similar to the Tracking Cohort. Participants from the Tracking cohort were randomly selected from the ten provinces of Canada and data were collected through telephone interviews. Three data points are accessible for analyses with a fourth wave available by the end of 2025. Data are available from the Canadian Longitudinal Study on Aging (www.clsa-elcv.ca) for researchers who meet the criteria for access to de-identified CLSA data. These data have also been linked to the Canadian Urban Environmental Health Research Consortium (CANUE) data set, which includes aggregated environmental data to assess urban health. Health administrative data will also be linked to the CLSA, including hospital, primary care, long-term care and pharmacological data at the individual level. Together, these data provide over a million data points to which AI analytic techniques could be employed.

## Conclusion

The integration of AI, especially the latest LLM, MLLM and AI sensor technologies, and advanced data analytics applied to resilience, offers a promising pathway to transform the landscape of brain health. Yet, there are many challenges and barriers to harnessing the potential of AI that require interdisciplinary collaborations, including merging knowledge from both applied and basic research. While the challenges are not trivial, there is potential for researchers and clinicians can develop more nuanced understandings of cognitive aging processes and more effectively intervene to enhance brain resilience across diverse populations. Employing a unified resilience model to this field offers a platform to forge new pathways into the growing field of prevention and treatment of ADRD. This proactive, predictive, and personalized approach is not just the future of brain health research; it is a necessary evolution to meet the growing complexities of aging populations worldwide.
